# Nanoporous Graphene Oxide-Based Quartz Crystal Microbalance Gas Sensor with Dual-Signal Responses for Trimethylamine Detection

**DOI:** 10.3390/s22249939

**Published:** 2022-12-16

**Authors:** Guangyu Qi, Fangfang Qu, Lu Zhang, Shihao Chen, Mengyuan Bai, Mengjiao Hu, Xinyan Lv, Jinglei Zhang, Zhenhe Wang, Wei Chen

**Affiliations:** 1 School of Agricultural Engineering and Food Science, Shandong University of Technology, Zibo 255049, China; 2 College of Mechanical and Electrical Engineering, Fujian Agriculture and Forestry University, Fuzhou 310002, China; 3 School of Food and Health, Zhejiang A&F University, Hangzhou 311300, China

**Keywords:** quartz crystal microbalance, nanoporous graphene oxide, gas sensor, dual signal, trimethylamine

## Abstract

This paper presents a straightforward method to develop a nanoporous graphene oxide (NGO)-functionalized quartz crystal microbalance (QCM) gas sensor for the detection of trimethylamine (TMA), aiming to form a reliable monitoring mechanism strategy for low-concentration TMA that can still cause serious odor nuisance. The synthesized NGO material was characterized by transmission electron microscopy, X-ray photoelectron spectroscopy, and Fourier transform infrared spectroscopy to verify its structure and morphology. Compared with the bare and GO-based QCM sensors, the NGO-based QCM sensor exhibited ultra-high sensitivity (65.23 Hz/μL), excellent linearity (R^2^ = 0.98), high response/recovery capability (3 s/20 s) and excellent repeatability (RSD = 0.02, n = 3) toward TMA with frequency shift and resistance. Furthermore, the selectivity of the proposed NGO-based sensor to TMA was verified by analysis of the dual-signal responses. It is also proved that increasing the conductivity did not improve the resistance signal. This work confirms that the proposed NGO-based sensor with dual signals provides a new avenue for TMA sensing, and the sensor is expected to become a potential candidate for gas detection.

## 1. Introduction

Trimethylamine (TMA), a typical natural degradation product of nitrogenous macromolecules in organisms, is widely used as the raw material and analytical reagent in pharmaceutical synthesis, pesticide manufacturing and the dye industry [1]. TMA is generally a colorless and toxic gas, which generates a fish-like odor at lower concentrations and an ammonia-like odor at higher concentrations [2]. Long-term inhalation exposure to trimethylamine may cause respiratory tract irritation, congestion and epithelial degeneration, even when it is present under a low chemical concentration [3,4,5]. TMA is also one of the effective indicators for the assessment of human health related to kidney disease, trimethylaminuria and respiratory disease [6]. In addition, TMA vapor may form an explosive mixture in the air and may burn or explode violently under open fire or high temperature [7]. Hence, the development of TMA gas sensors with high sensitivity and selectivity to meet different demands has crucial practical significance.

To date, various gas sensors for TMA detection have been developed with good sensing performance, including resistance sensors [8,9,10], colorimetric sensors [11,12,13,14], biosensors [15,16], kinetics methods [17] and spectral imaging [18,19,20]. However, these sensors generally have certain deficiencies, such as high operating temperature, poor stability and insufficient detection limit of TMA below its odor threshold [5,21]. Quartz crystal microbalance (QCM) sensors operate in accordance with gravimetric detection based on the piezoelectric effect, which has currently attracted intensive attention due to its ultra-high sensitivity, low energy consumption and easy modification [21,22,23,24,25,26,27]. The detection of trace TMA volatiles by coating sensing materials on the QCM sensor chips has proved to be an effective approach [28]. This approach focuses on increasing the conductivity and specific surface area of the sensing material since they are closely related to the interactions between analytes and materials. Graphene oxide (GO) nanosheets, as a two-dimensional material with only surface and no volume, have oxygen-containing functional groups on the surface and edge, which makes them a promising material for gas adsorption and desorption [29].

Recent studies have shown that chemically modified GO materials have a larger surface area, more obvious conductivity and better compatibility compared with bare GO material [30,31,32,33,34]. Furthermore, the existence of oxygen-containing groups also makes chemical modification easier. Based on chemically modified GO, the nanoporous structure of the sensor materials, such as steam-etching graphene and foam-like graphene, provides even larger sensing areas, which is beneficial for adsorbing more gas molecules. To the best of our knowledge, there are few reports about synthesizing the nanoporous functional-modified GO materials and coating them on a QCM sensor chip for the detection of TMA. Furthermore, general studies only analyze the resonant frequency signal of QCM, which decreases when the target analyte is adsorbed on the sensor surface. However, the resonance resistance signal is usually neglected, which also provides valuable analytical information. In this respect, the combined analysis of the frequency shift signal and resistance signal as a dual signal may generate a more comprehensive evaluation of the QCM sensing performance.

In this study, the nanoporous GO (NGO) material etched by hydrogen peroxide was synthesized by the hydrothermal method and characterized by spectral and imaging analysis techniques. Subsequently, the QCM sensors functionalized with GO and NGO nanomaterial were fabricated and compared to detect TMA gas, and the sensing mechanism was also discussed in detail. The sensitivity, selectivity and stability of the gas sensors were investigated based on the analysis of dual signals (i.e., frequency shift and resistance) across different TMA concentrations, which aimed to provide efficient technical methods enabling the detection of very low concentrations of TMA.

## 2. Materials and Methods

### 2.1. Reagents and Materials

The reagent 1-octen-3-ol (≈98%, CAS number: 3391-86-4) was purchased from Macklin Biochemical Co., Ltd. (Shanghai, China). Ammonia (NH_3_) (25–28%, CAS number: 7664-41-7) and hydrogen peroxide (H_2_O_2_) (≈30%, CAS number: 7722-84-1) were purchased from Shuangshuang Chemical Co., Ltd. (Yantai, China). Acetic acid glacial (≥99.5%, CAS number: 64-19-7) was purchased from Damao Chemical Reagent Factory Co., Ltd. (Tianjin, China). Ethyl acetate (≥99.5%, CAS number: 141-78-6) was purchased from Zhiyuan Chemical Reagents Co., Ltd. (Tianjin, China). Dimethylamine (DMA) (≈40%, CAS number: 124-40-3) and hexanal (≈97%, CAS number: 66-25-1) were obtained from Yien Chemical Technology Co., Ltd. (Shanghai, China). Trimethylamine (≈30%, CAS number: 75-50-3), formaldehyde (37–40%, CAS number: 50-00-0), acetone (≥99.5%, CAS number: 67-64-1), anhydrous ethanol (≥99.7%, CAS number: 64-17-5) and trisodium citrate dihydrate (CAS number: 6132-04-3) were purchased from Sinopharm Chemical Reagent Co., Ltd. (Shanghai, China). Gold (III) chloride trihydrate (HAuCl_4_·3H_2_O) (CAS number: 16961-25-4) was purchased from Sigma-Aldrich Trading Co., Ltd. (Shanghai, China). Graphene oxide (GO, ≈99% purity, CAS number: 7440-44-0) and silver nanoparticles (Ag NPs, 15 nm diameter, CAS number: 85-60-9) were provided by Nanjing XFNANO Materials Tech Co., Ltd. (Nanjing, China). The AT-cut 9 MHz quartz crystals with gold electrodes on each side were purchased from Renlu Crystal Co., Ltd. (Shenzhen, China). All reagents were of analytical grade and used as received without further purification. Deionized water was used throughout all experiments.

### 2.2. Synthesis of Nanoporous Graphene Oxide and Gold Nanoparticles (Au NPs)

NGO was synthesized by etching GO via hydrogen peroxide based on the hydrothermal method. Briefly, 4 mL of H_2_O_2_ aqueous solution (30%) was mixed with 10 mL of GO aqueous dispersion (2 mg mL^−1^). Then, the precursor solution was heated at 118 °C with mechanical stirring for 4 h to obtain the homogenous porous preforms. The as-prepared NGO was obtained by centrifuging and washing the porous preforms between triple and quintic to eliminate residual H_2_O_2_. Subsequently, the obtained NGO materials were re-dispersed in deionized water for further usage in modifying the QCM chip sensor. The Au NPs mechanically mixed with GO and NGO were prepared from HAuCl_4_·3H_2_O and trisodium citrate dihydrate. Under the condition of the water bath, 10 mL of 1 mM HAuCl_4_·3H_2_O and 25 mL of 1 mM trisodium citrate dihydrate solution were mixed evenly. During the addition process, it was necessary to stir the solution violently to prevent Au NPs from agglomeration to ensure the growth of it and finally obtain Au NPs [35].

### 2.3. Morphology Characterization of Sensor Materials

The morphologies of the GO/NGO sensor materials were observed to investigate the structural differences caused by the hydrogen peroxide etching processes and the porous preforms using a transmission electron microscope (TEM, WJGS-032, FEI Co., Ltd., Hillsboro, OR, USA). Spectroscopic characterization was performed to study the molecular changes in GO/NGO during the process of material synthesis using X-ray photoelectron spectroscopy (XPS, PHI Quan-tera II, Thermo Fisher Scientific, Waltham, MA, USA) and Fourier transform infrared spectroscopy (FT-IR, Nicolet5700, Thermo Nicolet Corporation, Madison, WI, USA).

### 2.4. Fabrication of GO/NGO-Based QCM Chip Sensors and Gas Sensing Measurements

The GO/NGO-based QCM chip sensors were fabricated using the drip coating method for highly sensitive detection of TMA. GO/NGO dispersed in deionized water was absorbed with a capacity of 2.5 μL each time via a pipette gun and then uniformly coated on one surface of the QCM chip in the headspace vial and repeated ten times. The fabricated GO/NGO-based QCM chip sensors were dried with an infrared lamp for 2 h to remove residual solvent. After stabilization for 12 h, the dry mass of GO and NGO coated on the surface of the QCM chip was 13.866 and 10.224 μg, respectively. All experiments were conducted under ambient temperature (23 °C) and stable humidity (40%) conditions to maintain the consistency of data collection. The measurement of temperature and humidity before each round of sample detection was implemented using the thermos hygrograph (HM34C, Vaisala, Finland).

According to the national standard (GB5009.228—2016), the nitrogen-containing gas deteriorated at 3 μmol. Ideally, 1 μmol TMA was put into a 50 mL gas bottle. In fact, equivalent liquid was inhaled and put into an 1158 mL gas bottle. According to the constant mass, the volume of the pure solution corresponding to 1 μmol TMA can be calculated as 1.96 μL by using the formula: V =$\;\frac{n*M}{\rho }$ (parameters in Table 1). Through this formula, any gas concentration required by the experiment can be configured.

**Figure 1:**
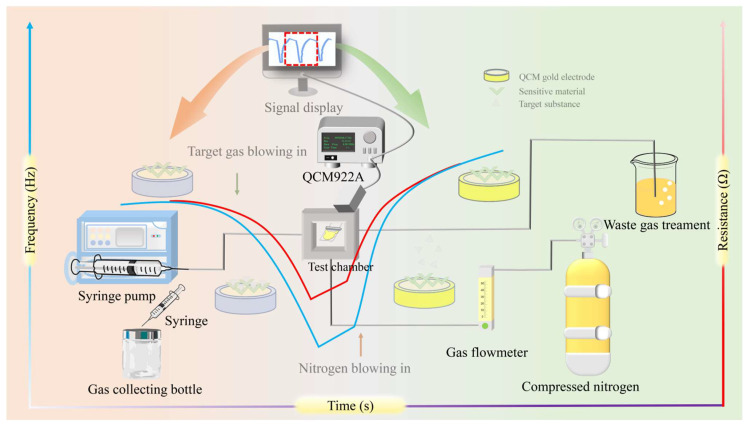
Schematic diagram of detection based on functionalized QCM sensor chip.

As for sample testing, the sensing properties of the sensor devices were measured with a laboratory-made measurement setup, as shown in Figure 1. TMA vapor was obtained by the static headspace sampling method and was tested with the prepared sensors to evaluate the sensing performance of the sensors, respectively. A certain volume of TMA solution was dropped into a 1.158 L reagent bottle, then retained at room temperature (RT) to let it completely evaporate for leaving overnight. DMA, NH_3_, formaldehyde, hexanal, acetic acid glacial, ethyl acetate, 1-octen-3-ol, anhydrous ethanol and acetone vapor were prepared by the same headspace sampling method, and the selectivity of the QCM sensors as researched. The analyte gas TMA (with a concentration of 1–10 μL/1158 mL) was sucked out of the collection bottle and injected into the CL6 (Negligible chamber volume) gas chamber at the speed of 10 mL/min through an automatic syringe during the sensing (response) measurement, which was aimed to test the adsorption of GO/NGO materials (modified on the surface of sensor chip) to the gases. Nitrogen controlled by a valve was injected into the chamber to wash the TMA gas for the purging (recovery) process, which was aimed to test the desorption of the sensor chip. Finally, the tail gas was treated with a beaker filled with water. The real-time sensor responses to the absorption and desorption of gases, including frequency change and resistance, were measured by the QCM platform (QCM922A, OA-CL6, Ametek, Philadelphia, PA, USA) for further experimental data analysis. In Figure 1, the blue line represents frequency, and the red line represents resistance. With the injection of the gas, gas molecules are adsorbed on the surface of the QCM gold electrode, and the quality of the gold electrode increases. This leads to a decline in the curve. When the gas injection is completed, the gas will be desorbed. This process will be accelerated by nitrogen injection.

## 3. Results and Discussion

### 3.1. Characteristics Analysis of GO/NGO

**Figure 2:**
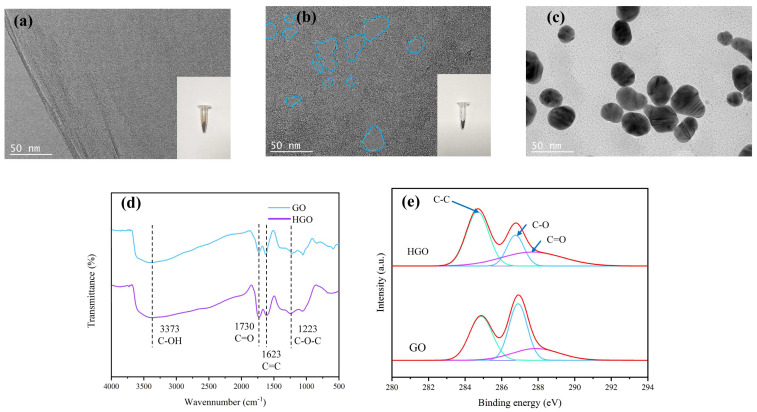
Characteristics analysis of GO/NGO. (**a**) TEM image (inserted panel shows synthesized powder) of GO, (**b**) TEM image (inserted panel shows synthesized powder) of NGO, (**c**) TEM image of Au NPs, (**d**) FT-IR spectra of GO and NGO, and (**e**) XPS spectra of GO and NGO.

As shown in Figure 2, the morphology and chemical structure of sensor materials GO/NGO were investigated by TEM, FT-IR and XPS analyses. The TEM-image in Figure 2a,b verified that the porous structure of NGO was successfully synthesized based on GO etched by hydrogen peroxide (The blue line of Figure 2b was etched porous). The unstable oxidized SP^3^ domain in the GO sheet was more active under the condition of oxidative chemical etching and finally formed nanopores surrounded by carboxylic acid functional groups [24]. Figure 2c shows the TEM picture of Au NPs. The Au NPs were relatively uniform, and the particle size was about 25 nm. The FT-IR spectra of GO and NGO are shown in Figure 2d. For both GO and NHO, the peak that appeared around 3373 cm^−1^ was the from the C-OH bond, the peak at 1730 cm^−1^ was attributed to the stretching vibration of the C=O bond, the peak located at 1623 cm^−1^ was assigned to the vibration of the C=C bond and the peak at 1223 cm^−1^ was formed by the stretching vibration of the C-O-C bond. These results indicated that GO and NGO had similar chemical functional groups, such as ether, epoxy, carboxylic acid and hydroxyl groups. For the high-resolution XPS spectra of GO and NGO depicted in Figure 2e, three peaks, located at 285, 287 and 288 eV, were attributed to the C-C, C-O and C=O bonds, respectively. The XPS measurement results confirmed that during chemical etching, the concentration of the carboxylic acid group increased, while the concentration of the epoxy group and hydroxyl group decreased [36]. In general, the TEM, FT-IR and XPS analyses were able to prove that NGO with nanoporous structure was successfully synthesized by etching GO via hydrogen peroxide.

### 3.2. Dynamic Responses of QCM (Bare and GO-Based) Sensor to Gas Vapors

**Figure 3:**
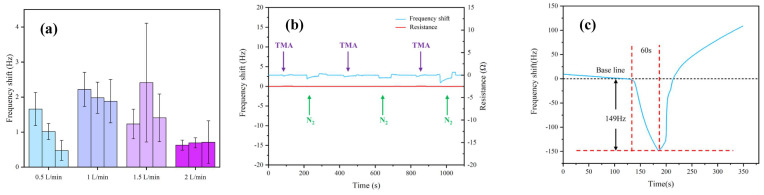
Dynamic responses of QCM sensor to gas vapors. (**a**) Influence of different nitrogen flow rates on the QCM sensor, (**b**) the bare QCM sensor to TMA, and (**c**) the GO-based QCM sensor to TMA and nitrogen.

The dynamic responses (i.e., frequency shift and resistance) of the QCM sensor chip under exposure to gas vapors (i.e., TMA and nitrogen) were depicted in Figure 3. In order to explore the influence of different flow rates of N_2_ on the modified sensitive material gold electrode, the following control experiments were performed. The N_2_ was entered into the chamber at four different intake speeds: 0.5, 1, 1.5 and 2 L/min. For comparison, the time is adjusted to ensure that the volume of gas entered the atmospheric chamber is fixed at 1.5, 2 and 2.5 L, respectively. Due to the small volume of the gas chamber, 1.5 L of N_2_ can replace all the indoor gases. Figure 3a shows that when the N_2_ flow rate was 2 L/min, the frequency fluctuation of the gold electrode was the smallest. Finally, according to the results of the control experiment, an appropriate N_2_ flow rate was chosen for the desorption of the detected gas and the sensitive material.

As shown in Figure 3b, the bare QCM sensor chip (without modified sensitive material on its surface) had a weak frequency shift response and no resistance response to TMA, indicating that frequency shift had a more active response than resistance and the bare sensor had practically no adsorption selectivity to TMA. To compare the sensing performance based on frequency shift, the single-cycle dynamic characteristics of the as-prepared GO-functionalized QCM sensor chip under exposure to TMA vapors with a concentration of 5 μL/1158 mL were measured (Figure 3c). The frequency of the GO-based QCM sensor shifted sharply (the discrepancy was 149 Hz) when the TMA vapors were introduced to the chamber for 60 s. The TMA in the gas chamber was then cleaned with nitrogen to restore the sensor’s frequency offset sharply to its original baseline value. A longer time was observed during the recovery phase compared to the response phase to ensure that TMA was completely discharged from the chamber, which recovered the re-adsorption capacity of the GO-based QCM to TMA. Table 2 lists the relevant parameters of some other TMA sensors.

### 3.3. Comparison of Sensing Characteristics between GO and NGO-Functionalized QCM Sensor

**Figure 4:**
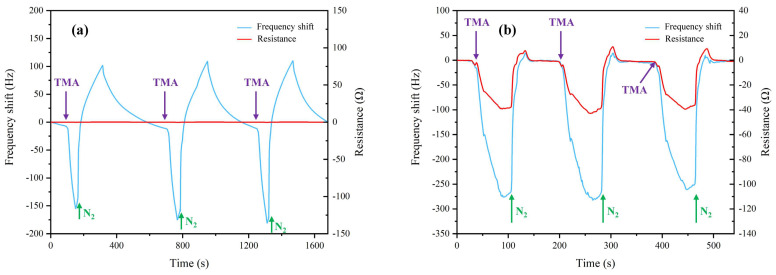
Sensing characteristics of QCM sensors to gas vapors. (**a**) The GO-based QCM sensor, and (**b**) the NGO-based QCM sensor.

In order to compare the sensing performances and verify the repeatability indexes of the GO/NGO-based QCM sensors, three-cycle assessments of the functionalized QCM sensor under exposure to TMA (with a concentration of 5 μL/1158 mL) and nitrogen (as control) were measured (as shown in Figure 4). The three-cycle frequency shift responses of the GO-based QCM sensor (as depicted in Figure 4a) had similar curve shapes, response amplitude, response time and recovery time, which demonstrated the repeatability of the sensor. However, this sensor had no resistance response to the change in different gas vapors, which indicated its lack of sensitivity. Compared with the frequency shift and resistance responses of the GO-based QCM sensor, the sensing performances of the NGO-based QCM sensor achieved higher frequency shift values (approximately 275 Hz) and more notable resistance responses (as shown in Figure 4b). Because nitrogen blowing was not an automatic injection, the duration was not completely consistent. Specifically for the resistance response of the NGO-based QCM sensor, it not only had a consistent response regularity with the frequency shift but also had a significant response to the switching between TMA and nitrogen. These results proved that the proposed NGO-based QCM sensor had a more sensitive gas-sensing ability and could be used for the absorptive detection of target gas TMA.

### 3.4. Sensing Performances of GO and NGO-Functionalized QCM Sensors for TMA Detection

**Figure 5:**
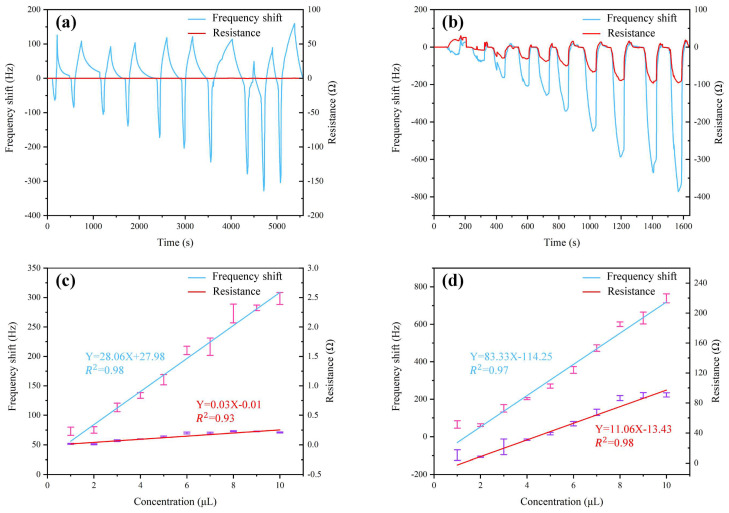
Sensing performances of QCM sensors for TMA detection. (**a**) Frequency and resistance response curves of the GO-based QCM sensor, (**b**) frequency and resistance response curves of the NGO-based QCM sensor, (**c**) response amplitude regression curves of the GO-based QCM sensor, and (**d**) response amplitude regression curves of the NGO-based QCM sensor.

Figure 5 illustrates the sensor performances to the increasing concentration of TMA (1–10 μL/1158 mL). Except for the different resistance responses between GO and NGO-functionalized QCM sensors, similar trends of frequency shift response with the increase in TMA concentration were observed in Figure 5a,b. The resistance curve of the first four hundred seconds in Figure 5b was not perfect. Under the same experimental conditions, the trend of the resistance curve was inconsistent with others, which might be caused by the unstable startup of the instrument. The response amplitude (lowest point) showed a negative linear correlation to the concentration of TMA. In this respect, the linear regression curves between TMA concentration and the GO/NGO-based QCM sensor response amplitudes are plotted in Figure 5c,d, respectively. The experimental data showed that the resistance response amplitude of the GO-based QCM sensor also had a linear correlation to TMA concentration within a minute scale interval, although it was difficult to be observed intuitively. The data followed the linear fittings with R2 values of 0.98 and 0.93 for frequency shift and resistance response amplitude of the GO-based QCM sensor, as well as 0.97 and 0.98 for those of the NGO-based QCM sensor, respectively. The results indicate that the NGO-based sensor possessed very high sensitivity and selectivity to TMA vapors, which demonstrated the feasibility and effectiveness of our proposed NGO-based QCM sensor chip in detecting TMA at low concentrations.

### 3.5. Selectivity of GO and NGO-Functionalized QCM Sensors to Different Gases

**Figure 6:**
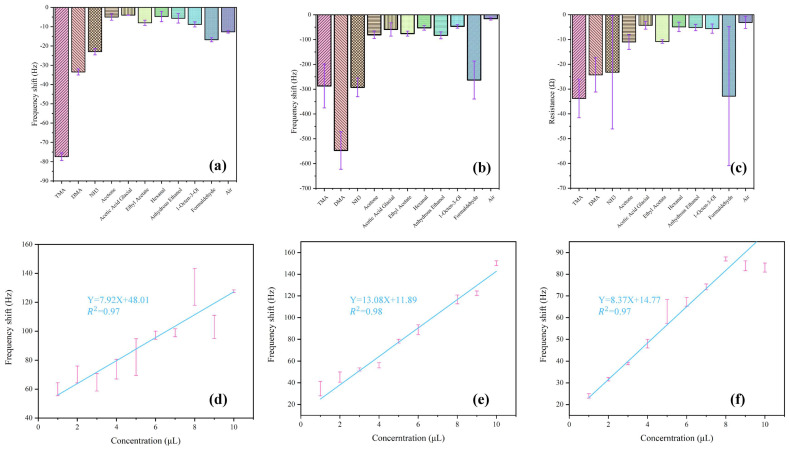
Selectivity of GO and NGO-functionalized QCM sensors to different gases. (**a**) Frequency response of the GO-based QCM sensor, (**b**) frequency response of the NGO-based QCM sensor, (**c**) resistance response of the NGO-based QCM sensor, (**d**) response amplitude regression curves of the NGO/Au Nps-based QCM sensor, (**e**) response amplitude regression curves of the NGO/Ag Nps-based QCM sensor, and (**f**) response amplitude regression curves of the GO/Ag Nps-based QCM sensor.

The adsorption selectivity of the GO/NGO-based sensors was investigated by performing multiple dynamic exposures to various chemical analytes, including TMA, DMA, NH_3_, acetone, acetic acid glacial, ethyl acetate, hexanal, anhydrous ethanol, 1-octene-3-ol, formaldehyde and air. Because the resistance response of the GO-based QCM sensor was weak, the frequency shift responses of the GO-based QCM sensor (Figure 6a) and the NGO-based QCM sensor (Figure 6b), and the resistance response of the NGO-based QCM sensor (Figure 6c) were selected for selective analysis. As shown in Figure 6a, the GO-based QCM sensor showed better interaction with TMA molecules compared to their ammonia counterparts (other nitrogenous gases), which could be due to the lower vapor pressure of TMA and better adsorption when it interacted with the sensing active layer materials [17]. Although the frequency shift response of the NGO-based QCM sensor showed relatively lower selectivity to TMA, that of the resistance response showed the highest selectivity. The QCM sensing results confirmed the high selectivity, sensitivity to TMA gas, and good stability and reliability of the proposed NGO-based QCM sensor, and the dual-band signal (i.e., frequency shift and resistance) analysis had a more comprehensive evaluation ability for QCM, which provided a new insight into the promising TMA sensor performances.

The dual signal verification of frequency and resistance was realized by detecting TMA with NGO material modified by the QCM gold electrode. Based on this consideration, Ag NPs and Au NPs were mechanically mixed with NGO to improve the response signal of resistance, respectively. However, this is not the case. As shown in Figure 6d–e, the composite materials only maintained a linear relationship with the frequency signal and had no impact on the resistance, and the frequency signal was lower than before. Figure 6f shows the comparison between GO and Ag NPs mechanical composite, which was still only linear in frequency and had no obvious linear relationship with the resistance. The resistance signal between NGO and TMA cannot be judged simply by its conductivity. This may be related to the frequency. When the frequency reaches a certain value, it will drive the resistance signal to change.

## 4. Conclusions

A highly sensitive and selective TMA detection sensor was fabricated using a low-cost drop-coating method consisting of a QCM platform coated with a nanoporous material synthesized by etching GO with hydrogen peroxide. To confirm the sensor materials, NGO was successfully synthesized based on the modification of GO. The morphology and chemical structure of GO and NGO were investigated by TEM, FT-IR and XPS analyses. The dual-band dynamic responses (i.e., frequency shift and resistance) of the QCM sensor chip under exposure to gas vapors proved that the NGO-based QCM sensor obtained an outstanding sensitivity towards TMA vapors compared with the bare sensor and the GO-based sensor. The proposed NGO-based QCM sensor showed a fast response and recovery times with highly reversible and repeatable dual-signal responses to different concentrations of TMA. For the change in resistance signal caused by TMA, the experimental results show that it cannot be simply attributed to the reason of conductivity. Moreover, this device also showed an acceptable high selectivity towards TMA over other nitrogenous gases according to the comprehensive analysis of dual-signal responses. This promising device, modified with new sensing materials, has the feasibility of being widely used in various chemical vapor detection and monitoring applications.

## Figures and Tables

**Table 1:** Formula parameters of the gas distribution.

Parameter	Meaning	Unit
V	Volume of liquid absorbed	μL
*n*	amount of substance	mol
*M*	molecular weight of the analyte	g/mol
ρ	density of the liquid sample	g/ml

**Table 2:** Comparison of different types of TMA sensors.

Sensor Type	Temperature Range	Sensing Parameter	Response Time	Recovery Time	Reference
QCM	RT	Frequency	3 s	15 s	This work
QCM	RT	Frequency	7 s	20 s	[2]
N-type Semiconductor	RT	Resistance	12 s	19 s	[3]
Metal oxide semiconductors	250 °C	Resistance	3 s	2 s	[9]
Metal oxides	133 °C	Resistance	90 s	6.8 min	[10]
